# Slow eccentric- versus concentric-focused resistance training in middle-aged and older women with knee osteoarthritis: A randomized trial

**DOI:** 10.1186/s13102-026-01882-8

**Published:** 2026-08-07

**Authors:** Ping Liu, Qian Zhao, Hengzhi Deng, Yao Lu, Lele Chang, Zhang Xuelin

**Affiliations:** 1 Faculty of Sports and Exercise Science, University of Malaya, Kuala Lumpur, Malaysia; 2 College of Physical Education, Qufu Normal University, Qufu, Shandong 273165 China; 3 Integrative Exercise Physiology Laboratory, Department of Physical Education, Jeonbuk National University, Jeonju, Jeollabuk-do 54896 South Korea

**Keywords:** Knee osteoarthritis, Slow eccentric-only training, Slow concentric-only training, Myofascial chains, Resistance training

## Abstract

**Objective:**

This randomized trial compared the short-term effects of slow eccentric-only resistance training (SET) and slow concentric-only resistance training (SCT), organized within a myofascial chain-based exercise framework, on pain, physical function, muscle strength, muscle mass, and joint range of motion in middle-aged and older women with knee osteoarthritis (KOA).

**Methods:**

In this randomized, assessor-blinded trial, 56 women with symptomatic KOA were assigned to SET (*n* = 28) or SCT (*n* = 28) at a university rehabilitation center. Both groups completed a 12-week supervised resistance-training program (2 sessions/week). Outcomes were assessed at baseline and post-intervention using the WOMAC index, muscle strength testing, body composition analysis, functional performance tests, and joint range-of-motion measurements. The trial was registered retrospectively (UMIN-CTR, UMIN000060649, 2026/02/11).

**Results:**

After 12 weeks, both SET and SCT showed significant within-group improvements in WOMAC pain and physical function, muscle strength, knee extensor torque, fat-free mass, lower-limb muscle mass, and selected functional performance measures (all *p <* 0.05). No significant between-group differences were detected for any outcome. The observed improvements primarily reflect within-group changes over time, and the results should not be interpreted as formal evidence of equivalence between the two training modes.

**Conclusion:**

Both SET and SCT were associated with within-group improvements in pain, physical function, muscle strength, and muscle mass over 12 weeks in middle-aged and older women with KOA, with no significant between-group differences. These findings suggest that both approaches are feasible short-term supervised resistance-training options in this population. Additional strategies targeting cardiorespiratory endurance and joint range of motion may be needed within a multimodal rehabilitation program.

**Trial registration:**

University hospital Medical Information Network Clinical Trials Registry (UMINCTR), UMIN000060649, retrospectively registered on 11/02/2026.

**Supplementary Information:**

The online version contains supplementary material available at https://doi.org/10.1186/s13102-026-01882-8.

## Introduction

Knee osteoarthritis (KOA), characterized by progressive cartilage degradation and joint dysfunction, is the most prevalent chronic joint disorder in adults, affecting over 365 million people globally and significantly reducing quality of life [1, 2]. Patients typically present with pain, stiffness, reduced range of motion (ROM), muscle weakness, and impaired physical function, leading to physiological and psychological burdens [3]. While no cure exists, exercise therapy remains a cornerstone of KOA management [3–5].

Resistance training is widely recommended for individuals with KOA because it may reduce pain and improve muscle performance and physical function [4, 6]. Previous studies suggest that both eccentric- and concentric-focused resistance exercise may be beneficial in KOA, although the magnitude and pattern of adaptation may vary across interventions and populations [7, 8]. However, direct comparisons between slow eccentric-only and slow concentric-only resistance training in women with KOA remain limited. Slow-velocity resistance training has been reported to promote muscle hypertrophy and may reduce the risk of excessive eccentric-related muscle damage or soreness, which may be relevant when prescribing exercise for individuals with KOA [9].

In addition to local knee-focused explanations, lower-limb symptoms and function in KOA may also be considered within a broader kinetic-chain framework [10–12]. Emerging evidence suggests that altered coordination, load distribution, and soft-tissue interactions across the lower limb may contribute to symptom persistence and functional limitation in KOA [13, 14]. In the present study, the myofascial chain concept was used solely as a theoretical framework for organizing multi-joint exercises, not as a directly measured mechanistic target. Because fascial properties were not assessed, any myofascial interpretation should be considered theoretical rather than confirmatory. The primary scientific gap addressed by this trial is the direct comparison between two muscle contraction modes within a specific clinical population, using a structured, supervised training protocol.

Therefore, the aim of this randomized trial was to compare the effects of slow eccentric-only and slow concentric-only resistance training on pain, physical function, muscle strength, muscle mass, and joint range of motion in middle-aged and older women with knee osteoarthritis. We hypothesized that both groups would demonstrate clinical and functional improvements, and we sought to investigate whether slow eccentric training produced a greater magnitude of response than slow concentric training.

## Methods

### Study design

A randomized, assessor-blinded, parallel-group trial was conducted between March and June 2024 at the Qufu Normal University Rehabilitation Center. The study protocol was approved by the Biomedical Ethics Committee of Qufu Normal University (Approval No. 2024 − 108). All participants provided written informed consent. This trial adheres to the CONSORT 2010 statement for reporting randomized trials. The trial was retrospectively registered at UMIN-CTR (UMIN000060649) on 11/02/2026 due to an administrative delay; prospective registration was not completed before participant enrolment. This limitation is explicitly addressed in the Discussion.

### Participants

#### Sample size calculation

Sample size was estimated using G*Power 3.1 based on the planned between-group comparison of the single primary outcome, the WOMAC pain subscale, assuming an effect size of 0.5 derived from our unpublished pilot work (*n* = 10 per group), with α = 0.05, power = 0.80, and an allocation ratio of 1. The minimum required sample size was 56 participants. To account for potential dropout, 62 women were initially recruited. Other outcomes, including muscle strength, were treated as key secondary outcomes for which the study was not primarily powered.

#### Inclusion criteria

(i) women aged > 50 years; (ii) knee osteoarthritis diagnosed according to the American College of Rheumatology criteria, with radiographic evidence of tibiofemoral osteophytes on X-ray and a Kellgren–Lawrence grade of I–III; (iii) knee pain on most days during the previous month; and (iv) a pain intensity score of ≥ 4 on an 11-point numeric rating scale (NRS; 0 = no pain, 10 = worst imaginable pain) during the previous week.

#### Exclusion criteria

(i) intra-articular injection in the affected knee within the previous 6 months; (ii) lower-limb surgery within the previous 12 months; (iii) neurological, autoimmune, or other systemic conditions that could affect mobility, pain perception, or safe participation in exercise training; (iv) severe lower-limb deformity interfering with training or assessment; (v) inability to understand or follow study instructions; and (vi) BMI > 35 kg/m². Only women were included in this study to improve sample homogeneity and reduce potential sex-related variability in symptom presentation and training response.

Participants were instructed to maintain their usual medication regimen throughout the study unless a medically necessary change occurred. Medication use, including type and frequency, was recorded at baseline and monitored during the intervention period. Participants were also asked to report any medication changes immediately.

### Randomization and blinding

Block randomization (block size of 4, no stratification) was implemented by an independent statistician who generated the random allocation sequence. Sequentially numbered, sealed, opaque envelopes were prepared by a research assistant not involved in participant enrolment or assessment. Envelopes were opened only after participant enrolment and completion of baseline assessment, ensuring allocation concealment. Outcome assessors remained blinded to group allocation throughout data collection. The success of blinding was not formally assessed.

### Intervention measures

#### Myofascial chain-based training protocol

Both groups completed a 12-week supervised resistance-training program, with two sessions per week. Each session consisted of three parts: (1) a 10-minute warm-up, (2) the main resistance-training exercises, and (3) a 5-minute cool-down. Total session duration ranged from approximately 60 to 90 min depending on participant tolerance, individualized rest periods, therapist-assisted movement execution, and symptom-based adjustment of exercise pace and load.

The warm-up consisted of 10 min of slow barefoot walking on a yoga mat. Participants were instructed to maintain slight knee flexion, gently lift the heel, and shift body weight toward the forefoot while walking at a controlled slow pace [15].

**Fig. 1:**
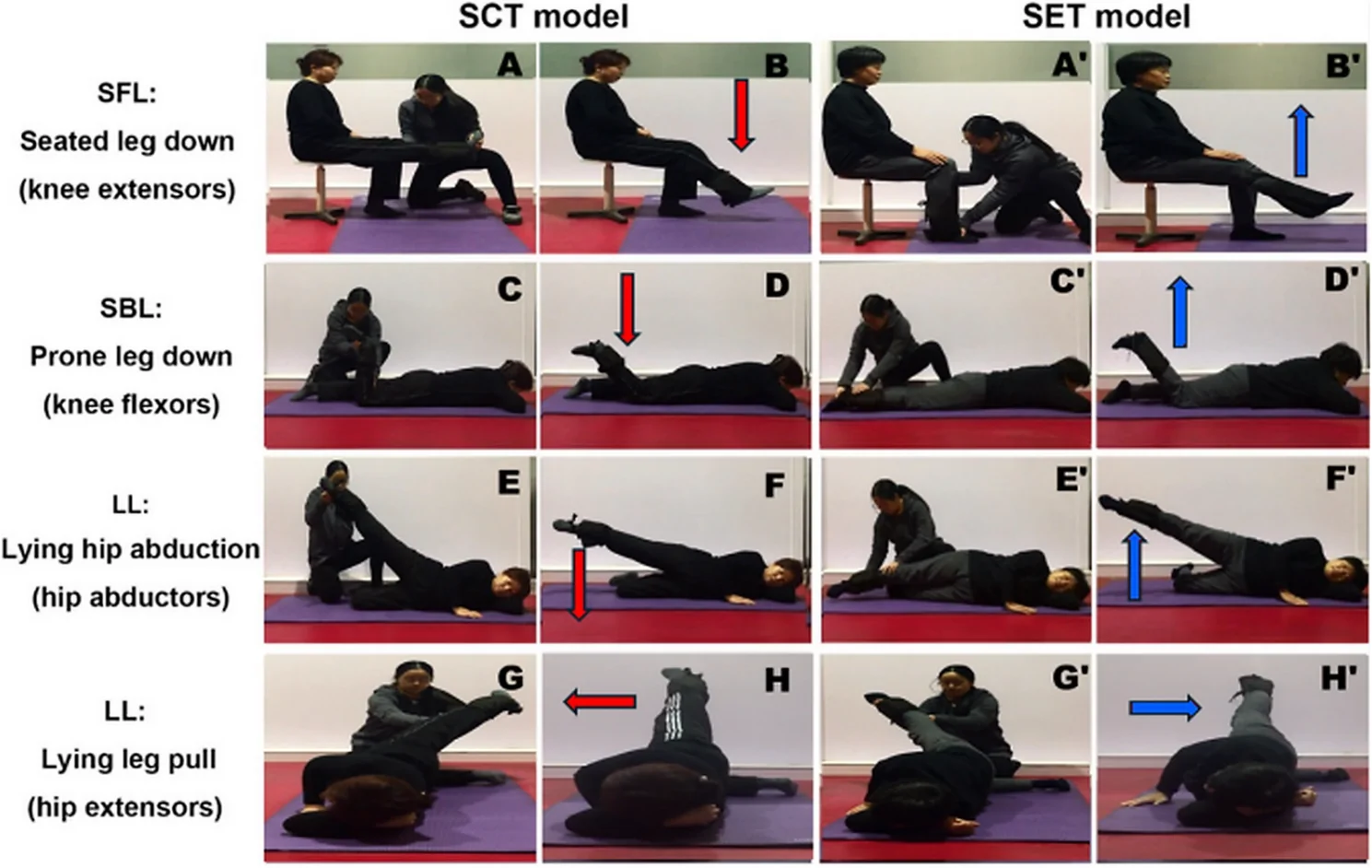
Slow eccentric-only training model and slow concentric-only training model based on myofascial chains. In the SCT model, the researcher completed the eccentric phase of the movement (panels **A, C, E, G**), whereas the participant independently and slowly performed the concentric phase (panels **B, D, F, H**). In the SET model, the researcher completed the concentric phase (panels **A′**, **C′**, **E′**, **G′**), whereas the participant independently and slowly performed the eccentric phase (panels **B′**, **D′**, **F′**, **H′**)

The main exercise program was organized according to a myofascial chain-based framework involving the superficial front line (SFL), superficial back line (SBL), and lateral line (LL) [10, 11]. The exercise sequence was as follows: SFL-related exercises included leg press, knee extension, and ankle dorsiflexion; SBL-related exercises included hamstring curl, hip extension, and plantarflexion; and LL-related exercises included hip abduction and trunk lateral flexion [15, 16]. This framework was used exclusively to organize multi-joint exercise selection, not as a directly measured mechanistic variable.In the SCT group, participants actively performed the concentric phase of each movement at the prescribed tempo, while the researcher provided the minimal manual assistance necessary during the eccentric phase to guide the return movement and reduce active eccentric effort. In the SET group, participants actively performed the eccentric phase at the prescribed tempo, while the researcher provided the minimal manual assistance necessary during the concentric phase to assist the return movement and reduce active concentric effort. The manual assistance provided by the researcher was not quantitatively measured, but it was limited to the minimum required to maintain the intended contraction-specific emphasis and safe execution. We acknowledge that unquantified manual assistance represents a potential source of bias, and this is discussed under Limitations. The contraction-specific training models for the SET and SCT groups are illustrated in Fig. 1. “Slow” was operationally defined as an active contraction duration of approximately 8–10 s per repetition. The supervising researcher controlled the tempo through verbal counting, and participants were continuously monitored to ensure adherence to the prescribed movement speed. All sessions were supervised by the research team. Exercise was paused or modified immediately if participants experienced discomfort, dizziness, excessive fatigue, or distress.

### Training parameters

Training intensity was initially set at 40–50% of estimated one-repetition maximum (1-RM) and was progressed to 60–80% 1-RM based on weekly 10-RM testing and individual tolerance [17, 18]. Volume was standardized at 3 sets×10 repetitions with 90 s rest between sets. Adherence to the prescribed contraction tempo, pain during sessions, rating of perceived exertion (RPE), and number of completed repetitions were recorded throughout the intervention (Table 1).

**Table 1:** Training parameters for SET and SCT groups

Component	SET group	SCT group
Contraction Type	Eccentric-only	Concentric-only
Velocity	8–10 s eccentric phase	8–10 s concentric phase
Intensity	Initial 40–50% 1-RM, 60–80% 1-RM*	
Volume	3 sets × 10 reps	
Rest	90 s between sets	
Progression criterion	Successful completion of prescribed reps with acceptable RPE and pain	
Monitoring	RPE, pain, tempo adherence, completed reps	

*Estimated 1-RM calculated from 10-RM using the Brzycki equation. Adjusted weekly based on 10-RM tests and symptom tolerance

### Outcome measures

Outcome measures were selected to capture complementary domains of KOA status. Pain (WOMAC pain subscale) was the pre-specified primary outcome. Muscle strength, body composition, physical performance, and joint mobility were designated as secondary outcomes to characterize broader changes. Multiple functional performance tests were included because each reflects a different aspect of functional capacity [19].

#### Primary outcome 

Knee pain was assessed using the paper-based simplified Chinese version of the Western Ontario and McMaster Universities Osteoarthritis Index (WOMAC, version 3.1) pain subscale (0–20) [20]. This version has demonstrated satisfactory reliability and validity in mainland Chinese KOA populations (ICC > 0.80).

#### Secondary outcomes

##### Physical Function

WOMAC physical function subscale (0–68) and WOMAC stiffness subscale (0–8).

##### Muscle strength

*Isometric knee extensor torque* was measured using the geometric vertical squat model described by Sato et al. [21]. Participants assumed a semi squat position on a 30° inclined board with the back against a wall. The more painful leg served as the supporting leg. Standardized verbal encouragement was provided during the 5 s maximal hold. Torque (N·m) = 0.678 × body weight (kg) × 0.246 × height (m) × sin(θ), where θ is the lower leg inclination angle. This method was chosen for its clinical feasibility and previously reported validity (ICC = 0.85–0.96). Participants were instructed to stop immediately if they experienced intolerable pain or instability. Lower-limb muscle strength was also assessed using a 10-repetition maximum (10-RM) testing protocol; the corresponding 1-RM values reported are estimated 1-RM values derived from the achieved resistance loads using the Brzycki equation [18, 22]. Single-leg testing was conducted for knee extensors, knee flexors, hip extensors, and hip abductors on the more painful side, while bilateral lower-limb strength was assessed using the double-leg leg press.

##### Body composition

Fat-free mass and lower-limb muscle mass (kg) were measured using a bioelectrical impedance analyzer (JW33ZEUS9.9, South Korea), which has been validated against dual-energy X-ray absorptiometry in older adults (ICC > 0.90). Participants were instructed to refrain from eating for 2 h prior and to consume water moderately.

##### Physical function performance

20-m Timed Walk Test (s), 6-minute Walk Test (m), Chair-Stand Test (time for 10 repetitions), and Step Test (repetitions in 15 s) [19].

##### Joint Range of Motion (ROM)

Hip internal and external rotation were assessed using a smartphone goniometer application (Protractor), which has shown acceptable validity and reliability [23]. Knee flexion and extension ROM were measured in the supine position as active range of motion; lower values indicate greater restriction.

### Statistical analyses

Data distribution was assessed using the Shapiro–Wilk test. Baseline between-group differences were assessed using independent t-tests or Mann-Whitney U tests as appropriate. For the primary outcome (WOMAC pain), a paired t-test was used to examine within-group change, and an independent t-test on change scores was used for the between-group comparison, with the significance level set at α = 0.05 (two-tailed). No Bonferroni correction was applied to the single primary outcome. Secondary outcomes were treated as exploratory variables, and their p-values were interpreted with caution in light of multiple comparisons. Effect sizes were calculated as Morris-corrected Cohen’s d for paired designs, accompanied by 95% confidence intervals where feasible. An intention-to-treat analysis was performed using the Last Observation Carried Forward (LOCF) method for the three participants lost to follow-up; a per-protocol sensitivity analysis produced consistent results. Recognizing the limitations of LOCF, we acknowledge this as a study limitation and recommend future use of mixed models or multiple imputation. All analyses were performed using SPSS version 26.0.

## Results

### Participant flow and baseline characteristics

**Fig. 2:**
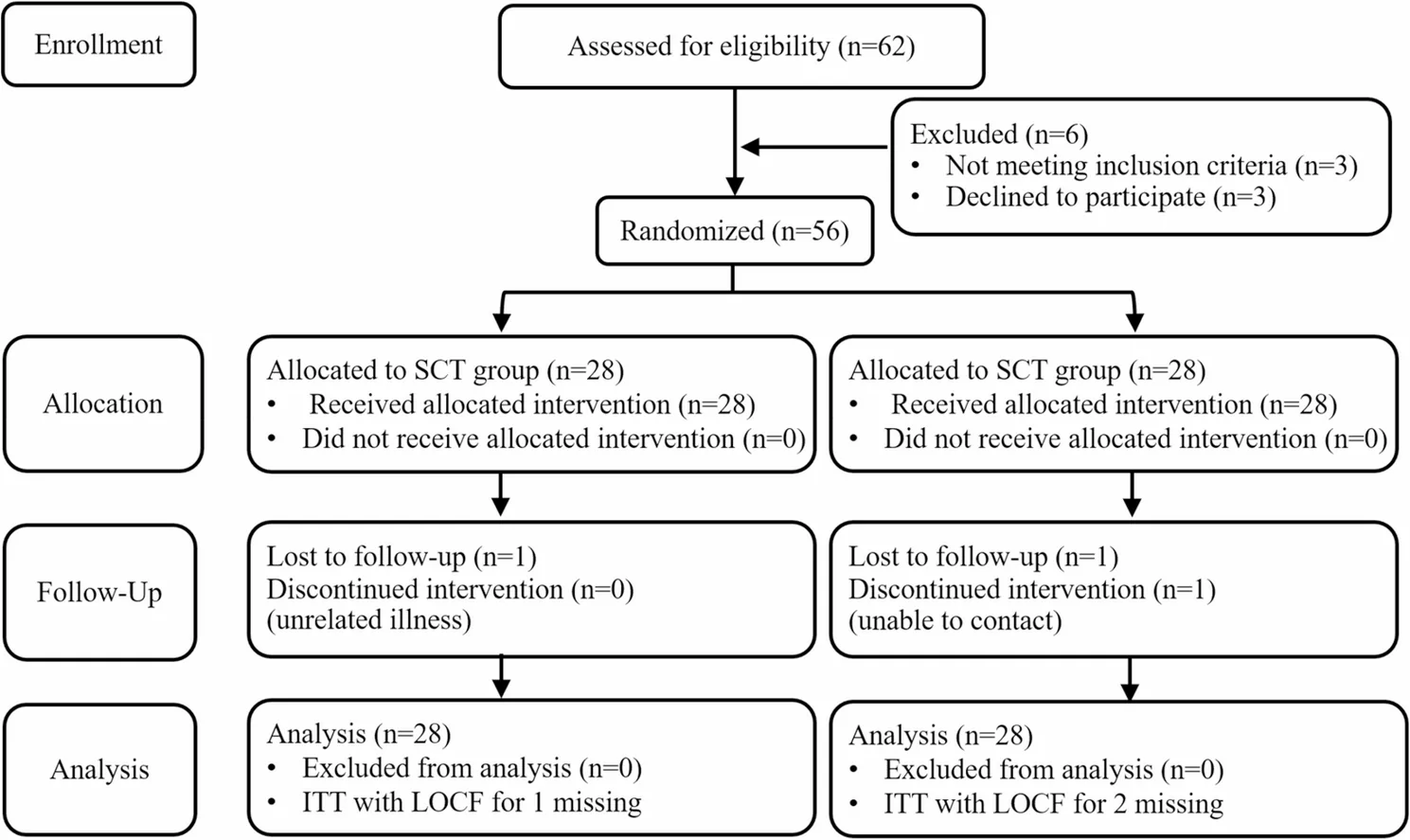
Flow of participants through the trial. Of 68 patients initially screened, 6 were not formally assessed, leaving 62 formally assessed for eligibility. Six were excluded (3 did not meet criteria, 3 declined). Fifty-six women were randomized. All received the allocated intervention. One participant in each group was lost to follow-up. An intention-to-treat (ITT) analysis using last observation carried forward (LOCF) was performed for all 56 participants

Of 68 patients screened, 62 were assessed for eligibility. Six were excluded (3 did not meet inclusion criteria, 3 declined participation). Fifty-six women with KOA were randomized (SET: *n* = 28; SCT: *n* = 28). Fifty-three participants (94.6%) completed the trial (SET: *n* = 27, SCT: *n* = 26). Two participants were lost to follow-up, and one discontinued due to an unrelated illness. An ITT analysis was performed using LOCF for the three missing cases. The CONSORT flow diagram has been revised for accuracy and is presented in Fig. 2.

Baseline demographic and clinical characteristics did not differ significantly between groups (Table 2, all *p* > 0.05). The mean age was 62.3 ± 6.7 years, and the majority had Kellgren–Lawrence grade 2 (*n* = 29) or 3 (*n* = 27). No participants with KL grade I were enrolled. Baseline WOMAC pain was 5.01 ± 2.0 (SET) and 4.67 ± 1.75 (SCT).

**Table 2:** Baseline characteristics of participants

Parameter	SET group (*n* = 28)	SCT group (*n* = 28)	*P* (between)
Age (y)	61.67 ± 6.81	63 ± 6.67	0.48
Height (m)	1.53 ± 0.21	1.6 ± 0.06	0.11
Weight (kg)	62.47 ± 11.67	66.23 ± 11.7	0.24
BMI (kg/m^2^)	26.8 ± 5.05	25.83 ± 4.94	0.48
KL grade (2/3), n	15 / 13	14 / 14	0.79
Symptom duration (months)	48.5 ± 32.1	52.3 ± 28.9	0.65
Affected side (left/right/bilateral)	5/8/15	6/9/13	0.82
Analgesic use, n (%)	12 (43%)	14 (50%)	0.59
WOMAC Pain (0–20)	5.01 ± 2.0	4.67 ± 1.75	0.55
WOMAC Function (0–68)	20.33 ± 3.31	21.17 ± 3.92	0.42

Values are mean ± SD unless otherwise noted

*﻿KL* Kellgren-Lawrence

### Primary outcomes

#### WOMAC pain 

After 12 weeks, both groups showed significant within-group reductions in WOMAC Pain (SET: Δ = -2.67, 95% CI [-3.6, -1.8], *p =* 0.015, d_rm_ = -1.89; SCT: Δ = -2.84, 95% CI [-3.8, -1.9], *p =* 0.026, d_rm_ = -2.23). These reductions exceeded the established Minimal Clinically Important Difference (MCID) of 2.0 points for WOMAC Pain. The between-group comparison revealed no significant difference (*p =* 0.516).

### Secondary outcomes

All secondary outcomes are presented in Table 3.

**Table 3:** Changes in outcome measures after 12 weeks

Outcome		Group	Baseline	12 weeks	Δ	95%CI(Δ)	d_rm_	*p* ^1^ (within)	*P* ^2^ (between)
Primary									
WOMAC	Pain (0–20)	SET	5.01 ± 2.0	2.33 ± 1.53	-2.67	-3.6, -1.8	-1.89	**0.015**	0.516
		SCT	4.67 ± 1.75	1.83 ± 0.75	-2.84	-3.8, -1.9	-2.23	**0.026**	
Secondary									
WOMAC	Stiffness (0–8)	SET	2.50 ± 1.23	1.02 ± 1.01	-1.48	-2.2, -0.8	-0.98	0.373	0.659
		SCT	1.33 ± 1.51	0.67 ± 1.03	-0.66	-1.3, 0.0	-0.48	0.102	
	Physical function (0–68)	SET	20.33 ± 3.31	15.23 ± 2.82	-5.10	-6.6, -3.6	-2.01	**0.001**	0.601
		SCT	21.17 ± 3.92	17.33 ± 5.89	-3.84	-5.4, -2.3	-1.28	**0.001**	
Muscle mass	Fat-free mass (kg)	SET	37.43 ± 3.03	39.08 ± 3.35	+ 1.65	0.9, 2.4	0.53	**0.020**	0.346
		SCT	40.75 ± 5.22	41.63 ± 5.36	+ 0.88	0.2, 1.6	0.17	**0.017**	
	lower limb muscle mass (kg)	SET	13.07 ± 0.95	13.98 ± 1.32	+ 0.91	0.5, 1.3	0.79	**0.008**	0.516
		SCT	13.52 ± 1.59	14.47 ± 1.21	+ 0.95	0.6, 1.4	0.69	**0.009**	
Physical function	Chair- stand test (s)	SET	27.25 ± 9.86	17.28 ± 2.03	-9.97	-15.1, -4.8	-1.07	**0.031**	0.104
		SCT	27.50 ± 10.55	22.77 ± 7.25	-4.73	-8.6, -0.9	-0.47	**0.049**	
	Step test (times)	SET	14.67 ± 2.81	19.17 ± 2.14	+ 4.50	1.2, 7.8	1.72	**0.021**	0.812
		SCT	17.33 ± 4.41	18.67 ± 4.55	+ 1.34	0.1, 2.6	0.31	**0.043**	
	20-meter timed walk test (s)	SET	17.07 ± 1.88	16.23 ± 1.69	-0.84	-1.7, 0.0	-0.48	0.058	0.158
		SCT	16.05 ± 2.36	14.59 ± 2.02	-1.46	-2.9, 0.0	-0.67	0.051	
	6-minute walk test (m)	SET	443.45 ± 58.48	486.31 ± 55.15	+ 42.86	-12, 98	0.75	0.210	0.782
		SCT	486.56 ± 85.28	498.73 ± 91.97	+ 12.17	-35, 59	0.14	0.222	
Joint ROM	Hip internal rotation (°)	SET	18.53 ± 2.71	31.01 ± 8.31	+ 12.47	7.8, 17.2	4.62	**0.011**	0.219
		SCT	25.42 ± 7.07	36.6 ± 6.35	+ 11.18	6.2, 16.1	1.57	**0.022**	
	Hip external rotation (°)	SET	18.52 ± 4.13	26.83 ± 8.82	+ 8.31	-2.4, 19.0	1.25	0.125	0.871
		SCT	18.22 ± 6.53	26.12 ± 5.76	+ 7.90	-1.9, 17.7	1.45	0.101	
	Knee flexion (°)	SET	53.61 ± 10.42	42.47 ± 12.44	-11.14	-22.5, 0.2	-1.17	0.093	0.980
		SCT	52.81 ± 13.31	42.32 ± 6.68	-10.49	-21.9, 0.9	-1.01	0.195	
	Knee extension (°)	SET	166.43 ± 4.91	167.77 ± 5.29	+ 1.34	-1.5, 4.2	0.27	0.218	0.142
		SCT	167.93 ± 4.47	172.65 ± 4.48	+ 4.72	-1.8, 11.2	1.05	0.142	
Muscle strength	Knee extensor 1-RM (kg)	SET	7.65 ± 1.31	10.58 ± 1.66	+ 2.93	2.0, 3.9	1.96	**0.001**	0.533
		SCT	7.53 ± 1.74	9.93 ± 0.64	+ 2.40	1.5, 3.3	1.83	**0.001**	
	Knee flexor 1-RM (kg)	SET	5.37 ± 1.28	6.95 ± 1.25	+ 1.58	0.9, 2.2	1.25	**0.006**	0.946
		SCT	5.52 ± 1.61	6.87 ± 1.48	+ 1.35	0.7, 2.0	0.87	**0.005**	
	Hip abductor 1-RM (kg)	SET	4.67 ± 1.05	5.57 ± 0.92	+ 0.90	0.2, 1.6	0.85	**0.050**	0.629
		SCT	4.98 ± 1.06	5.52 ± 0.50	+ 0.54	-0.1, 1.2	0.85	**0.050**	
	Hip extensor 1-RM (kg)	SET	4.43 ± 0.83	5.26 ± 0.51	+ 0.83	0.4, 1.3	1.19	**0.001**	0.687
		SCT	4.51 ± 1.27	5.53 ± 1.11	+ 1.02	0.3, 1.7	0.87	**0.038**	
	Double-Leg 1-RM (kg)	SET	88.75 ± 5.22	116.63 ± 5.36	+ 27.88	24.5, 31.2	4.14	**0.012**	0.503
		SCT	87.43 ± 3.03	112.18 ± 3.58	+ 24.75	21.9, 27.6	6.33	**0.015**	
Torque	Knee extensor torque (N*m)	SET	616 ± 57	842 ± 65	+ 226	160, 292	2.85	**0.026**	0.163
		SCT	620 ± 96	836 ± 65	+ 216	143, 289	1.66	**0.017**	

Values are mean ± SD. Bold indicates statistical significance (*p <* 0.05). The WOMAC Pain subscale was the single primary outcome. All secondary outcomes are presented with nominal p-values and should be interpreted cautiously due to multiple comparisons. Effect size was calculated as d_rm_ = [(M_post_ - M_pre_) / SDpre] × √[2(1-r)], where r was the estimated pre–post correlation (0.6–0.7). Interpretation: 0.2 = small, 0.5 = medium, 0.8 = large, ≥ 1.2 = very large, ≥ 2.0 = huge [24]. MCID for WOMAC Pain = 2.0 points [25]; both groups exceeded this threshold. MCID for WOMAC Function = 6.0 points; observed improvements approached but did not reach this threshold. The large effect sizes for strength measures reflect within-group responsiveness in previously untrained older women

*CI* Confidence interval, *SET* Slow eccentric-only training, *SCT* Slow concentric-only training

#### WOMAC function and stiffness 

WOMAC physical function improved significantly within both groups (SET: Δ = -5.10, 95% CI [-6.6, -3.6], *p =* 0.001, d_rm_ = -2.01; SCT: Δ = -3.84, 95% CI [-5.4, -2.3], *p =* 0.001, d_rm_ = -1.28), although the observed improvements approached but did not reach the MCID of 6.0 points. WOMAC stiffness did not change significantly in either group (both *p >* 0.05).

#### Muscle strength

Robust within-group increases were observed across all 1-RM measures. Double-leg 1-RM increased by 27.9 kg in SET (95% CI [24.5, 31.2]) and by 24.8 kg in SCT (95% CI [21.9, 27.6]), corresponding to very large effect sizes (d_rm_ = 4.14 and 6.33, respectively). Single-leg knee extensor 1-RM improved by 2.9 kg (SET) and 2.4 kg (SCT); knee flexor, hip abductor, and hip extensor 1-RM also increased significantly (all *p <* 0.05, Table 3). Isometric knee extensor torque improved by 226 N·m in SET and 216 N·m in SCT (both *p <* 0.05). No between-group differences were detected for any strength outcome.

#### Body composition 

Both groups showed modest but significant gains in fat-free mass (SET: +1.65 kg; SCT: +0.88 kg) and lower-limb muscle mass (SET: +0.91 kg; SCT: +0.95 kg) (all *p <* 0.05), with no between-group differences.

#### Physical function performance

Chair-stand time decreased significantly in both groups (SET: −10.0 s; SCT: −4.7 s), and step-test repetitions increased (SET: +4.5; SCT: +1.3) (all *p <* 0.05). In contrast, neither the 20-m timed walk nor the 6-minute walk test improved significantly (all *p >* 0.05; Table 3).

#### Joint range of motion 

Hip internal rotation increased significantly by approximately 11–12° in both groups (*p <* 0.05). No significant within- or between-group changes were observed for hip external rotation or knee flexion/extension ROM (all *p >* 0.05). The small numerical decrease in knee flexion was not statistically significant and likely reflects measurement variability.

No significant between-group differences were detected for any secondary outcome. These results should be interpreted cautiously given the exploratory nature of the secondary analyses and the absence of correction for multiple comparisons. The pattern of improvement was consistent across both groups, and the absence of between-group differences does not constitute formal evidence of equivalence.

## Discussion

### Key findings and context

This randomized trial found that 12 weeks of both slow eccentric-only and slow concentric-only resistance training were associated with within-group improvements in pain, physical function, muscle strength, and muscle mass in middle-aged and older women with KOA. No significant between-group differences were detected for any outcome. These results do not demonstrate superiority of one contraction type over the other, nor should they be interpreted as formal statistical equivalence. Rather, they suggest that both approaches, when delivered within a structured, supervised program, can yield comparable short-term clinical benefits.

A parsimonious interpretation of the similar gains is that both groups shared the most influential training characteristics: slow, controlled multi-joint movements, regular supervision, progressive overload, and a high degree of neuromuscular engagement [16, 26]. These shared features likely drove the majority of the observed adaptations, overriding any potential differential effects of contraction mode.

### Mechanisms underlying improvement

The clinically meaningful reduction in WOMAC pain (exceeding the 2.0-point MCID) is likely attributable to a combination of increased lower-limb muscle strength, improved joint stability, and enhanced load distribution across the knee joint [27–29]. Gains in muscle strength and fat-free mass, observed consistently across both groups, enhance the joint’s force-generating capacity and shock absorption capabilities, which can directly reduce nociceptive mechanical stress [6]. The improvements in chair-stand and step test performance further reflect enhanced functional neuromuscular control.

While the exercise program was organized around a myofascial chain framework, we acknowledge that this framework served only as a tool for exercise selection. We did not measure fascial stiffness, intermuscular force transmission, or dynamic joint kinematics. Therefore, any interpretations invoking myofascial chain adaptations remain theoretical and are not confirmed by our data. The observed benefits are more directly explained by established mechanisms of strength gain and neural adaptation [8, 26].

### Selectivity of effects

WOMAC stiffness did not change, consistent with evidence that self-reported stiffness is the least responsive domain to exercise in KOA and may reflect structural joint changes [3]. Neither the 20-m timed walk nor the 6-minute walk test improved significantly, underscoring the principle of training specificity: slow-velocity resistance training primarily enhances maximal force, not gait speed or cardiorespiratory endurance [30]. The lack of improvement in knee flexion/extension ROM likely reflects the irreversible structural constraints of established KOA (e.g., osteophytes, capsular fibrosis) [3]. The small numerical decrease in knee flexion observed in some participants was not statistically significant and may represent measurement variability; it should not be interpreted as worsening.

The significant increase in hip internal rotation aligns with the loading of hip musculature involved in the multi-joint exercises. However, greater internal rotation is not uniformly beneficial, as it could influence dynamic knee alignment. Without kinematic data, the clinical implication of this change remains uncertain and should not be overstated.

### Comparison with literature and clinical implications

Our finding of comparable benefits between eccentric and concentric training contrasts with some studies suggesting an advantage for eccentric work [7], but aligns with reviews indicating equivalent strength gains when total work is matched [8, 30]. The slow movement tempo (8–10 s per phase) likely equalized the total mechanical and metabolic stimulus between the two modes.

Clinically, this is encouraging: the choice between SET and SCT can be guided by patient preference, tolerance, and equipment availability, without concern for diminished short-term efficacy. SCT may be a more comfortable starting point for patients apprehensive about post-exercise soreness [9], whereas SET could be favored for those with concurrent tendinopathy [8]. However, since we did not systematically monitor pain during individual sessions, this suggestion remains a clinical hypothesis. Regardless of the contraction mode selected, our findings confirm that resistance training alone does not adequately address walking endurance or joint mobility, reinforcing the need for multimodal rehabilitation programs that include aerobic and mobility components [30].

### Limitations

Several limitations beyond those already mentioned must be considered. First, the trial was registered retrospectively due to an administrative oversight; prospective registration is the methodological standard, and this deviation should be weighed when considering the strength of the evidence. Second, the absence of a no-exercise or usual-care control group prevents isolation of the treatment effect from the natural course of symptoms or placebo effects. Third, manual assistance during exercise was not quantified, which may have introduced variability in the actual training load between participants and groups. Fourth, medication use was recorded but not standardized, representing a potential confounder for pain outcomes. Fifth, the 12-week duration precludes conclusions about long-term maintenance, and no follow-up was conducted. Sixth, the use of LOCF for missing data is a suboptimal approach that may introduce bias. Seventh, multiple secondary outcomes were tested without formal correction, increasing the risk of type I error for exploratory findings. Finally, the sample size was calculated only for WOMAC pain; the study was not powered for formal equivalence testing, and the absence of between-group differences should not be interpreted as evidence of equivalence.

## Conclusion

Twelve weeks of supervised slow eccentric-only and slow concentric-only resistance training, organized around a myofascial chain-based exercise framework, were both associated with significant within-group improvements in pain, physical function, muscle mass, and strength in middle-aged and older women with knee osteoarthritis. No significant between-group differences were observed, suggesting that both modalities are feasible short-term options. However, these interventions alone did not produce significant improvements in knee range of motion or cardiorespiratory endurance over the 12-week period, highlighting the need for multimodal rehabilitation programs. The results should be interpreted as evidence of comparable short-term response, not of formal equivalence.

## Supplementary Information

Supplementary Material 1

## Data Availability

The datasets used and/or analysed during the current study are available from the corresponding author on reasonable request.
